# Use of Statins in Kidney Transplant Recipients in Norway

**DOI:** 10.3390/ijerph19031370

**Published:** 2022-01-26

**Authors:** Marit Rønning, Vidar Hjellvik, Solveig Sakshaug, Hege Salvesen Blix, Karsten Midtvedt, Anna Varberg Reisæter, Hallvard Holdaas, Anders Åsberg

**Affiliations:** 1 Department of Drug Statistics, Norwegian Institute of Public Health, 0213 Oslo, Norway; maritroenning@gmail.com (M.R.); solveigsaks@gmail.com (S.S.); 2 Department of Chronic Diseases and Ageing, Norwegian Institute of Public Health, 0213 Oslo, Norway; Vidar.Hjellvik@fhi.no; 3 Department of Pharmacy, The Faculty of Mathematics and Natural Sciences, University of Oslo, 0316 Oslo, Norway; anders.asberg@farmasi.uio.no; 4 Department of Transplantation Medicine, Oslo University Hospital, Rikshospitalet, 0424 Oslo, Norway; kmidtved@ous-hf.no (K.M.); areisate@ous-hf.no (A.V.R.); hholdaas@ous-hf.no (H.H.); 5 Norwegian Renal Registry, Oslo University Hospital, Rikshospitalet, 0424 Oslo, Norway

**Keywords:** kidney transplant recipients, cardiovascular disease, statins, prescriptions, medication appropriateness, medication adherence, pharmacoepidemiology

## Abstract

Kidney transplant recipients (KTRs) experience increased risk of cardiovascular disease. Guidelines recommend HMG-CoA reductase inhibitor (statin) therapy when tolerated. We aimed to study changes in the prescription of statins and patients’ adherence to treatment over time. A population-based observational study utilizing linked data from the Norwegian Renal Registry (national coverage of 99.9%) and the Norwegian Prescription Database was performed. Data from a total of 2250 first KTRs were included (mean age—54 years, 69% men). Dispensed prescriptions of statins and immunosuppressants for the period 2004–2016 for all first KTRs engrafted in the period 2005–2015 were analyzed. Seventy-two percent received statins the first year after kidney transplantation and the proportion increased with age. The proportion receiving a statin varied according to the time frame of transplantation (77% in 2005–2010 vs. 66% in 2012–2015). Among new users of statins, 82% of the patients were adherent both the second and third year after kidney transplantation, while the corresponding figure for those already receiving statins before transplantation was 97%. Statin continuation rates in KTRs were high. In conclusion, our findings show a slightly lower overall proportion of patients receiving statins after kidney transplants than the national target level of 80%. The proportion of statin users increased with the age of the KTRs but showed a decreasing trend as time progressed.

## 1. Introduction

Kidney transplant recipients (KTRs) have a significantly higher risk of cardiovascular disease (CVD) compared to the general population [1] and this remains their leading cause of death [2,3,4,5]. Immunosuppressive therapies, especially the calcineurin inhibitors (e.g., cyclosporine (CsA) and tacrolimus (Tac)) and the mTOR inhibitors (e.g., sirolimus and everolimus) may induce post-transplant hypercholesterolemia via dense lipoproteins that are especially atherogenic. This is believed to be a main contributor to the increase in risk of CVD [6,7,8]. Current guidelines recommend HMG-CoA reductase inhibitor (statin) therapy to all adult KTRs (excluding fertile/pregnant females), irrespective of plasma lipid levels, provided the therapy is tolerated [5,9]. This is primarily based on data from the ALERT trial, investigating the effects of fluvastatin in 2102 KTRs with a total cholesterol level between 4–9 mmol/L at inclusion, showing protective effects on cardiac endpoints [10]. However, the tolerability of statin therapy in this population was limited in the pre-tacrolimus period by a high incidence of myopathy and rhabdomyolysis due to the pharmacokinetic interaction between CsA and statins [11]. This interaction induced increased systemic exposure of statins, eventually limiting their use to either fluvastatin or pravastatin in combination with CsA. With the introduction of Tac this has changed. The challenges of drug interactions are no longer the limiting factor and the probability of finding a tolerable drug combination has increased [12,13,14]. 

In line with current international guidelines [15], the national guidelines in Norway recommend starting statin therapy (fluvastatin) 2–3 months after kidney Tx. In patients experiencing side effects while using a statin, alternative statins should be tested before withdrawing the treatment. As a result of this, one of the quality indicators in the Norwegian Renal Registry is applying statin treatment for at least 80% of all KTRs. Annual data from the Norwegian Renal Registry indicate an increase in statin use following the publication of the ALERT study in 2003, stabilizing slightly below the 80% target level [16].

The aim of this study is to elucidate use of statins in KTRs by analyzing statin prescription dispensing data. We have focused on statin treatment before and after kidney transplantation and evaluated differences in use according to age, sex and changes in prescription and adherence over time. In addition, we studied inter-country differences in statin treatment and adherence to treatment over time.

## 2. Materials and Methods

This is a population-based observational study in KTRs based on linked data from the Norwegian Renal Registry and the Norwegian Prescription Database (NorPD). 

### 2.1. The Norwegian Renal Registry

The Norwegian Renal Registry is a national quality register for patients on renal replacement therapy. The registry includes all KTRs in Norway from the start of the transplant program in 1967. All renal units in Norway (*n* = 25) annually report central demographic, quality and outcome data to the registry. Based on annual crosschecks, the number of units reporting to the registry is considered comprehensive [16]. In our study, the following data were used from the registry: patient unique identifiers (encrypted), dates of transplantation (Tx) and dates of graft loss. 

### 2.2. The Norwegian Prescription Database (NorPD)

NorPD was established in 2004 and covers the total population in Norway. Since January 2004, Norwegian pharmacies electronically reported all dispensed prescriptions to the registry. NorPD contains information regarding all drugs prescribed and dispensed to individuals living outside institutions [17]. Drugs dispensed during hospital stays are not included. In NorPD all drugs are classified according to the Anatomical Therapeutic Chemical (ATC) classification system, and the amount of each drug dispensed per prescription is measured in Defined Daily Doses (DDDs) [18]. The following data were retrieved from NorPD for each recorded dispensing: patient unique identifier (encrypted), age, sex, county of residence, date the prescription was dispensed and drug information (ATC code and number of DDDs dispensed). 

All dispensed prescriptions of statins (ATC group C10AA-HMG CoA reductase inhibitors and C10BA-HMG CoA reductase inhibitors combined with ezetimibe) and immunosuppressants (ATC group L04A) were retrieved from NorPD for the period 2004–2016. Statins are considered a first-line treatment and represent 95% of all prescriptions and 96% of all DDDs for hypercholesterolemia in Norway (2016) [17]. Other drugs for hypercholesterolemia are mainly prescribed for severe and familial hypercholesterolemia. The aim was to elucidate use of statins in line with current guidelines, thus other drugs for hypercholesterolemia were not included in the analysis.

### 2.3. Study Population

The total study population included all patients receiving a first kidney transplant (living or deceased donor) in the period 2005–2015, at an age of 16 years or older, who were alive with a functioning first kidney allograft 365 days after transplantation and who had at least one prescription of immunosuppressants dispensed during 1–365 days after Tx. In analyses involving follow-up beyond 365 days after Tx, patients were censored at death or graft loss. In these analyses, only patients receiving a first kidney transplant between 2005–2013 (adherence analysis) and 2005–2012 (initiation of statin 2, 3 or 4 years after Tx) were included in order to achieve the necessary follow up time.

The total study population was divided into four groups according to use of statins one year (364–0 days) before and one year (1–365 days) after Tx. Previous users were defined as patients having at least one prescription of statins filled the year before Tx and no prescriptions the year after. Recurrent users were defined as patients having filled at least one prescription of statins the year before Tx and at least one prescription the year after. New users were defined as patients having no filled prescriptions of statins the year before Tx and at least one prescription filled the year after. Non-users were defined as patients with no filled prescriptions of a statin in the year before or in the year after Tx. New users and recurrent users were defined as two-year adherent users if they had a statin dispensed in the second year (366–730 days) after Tx and as three-year adherent users if they had additional statins dispensed in the third year (731–1095 days) after Tx. Previous users and non-users were defined as two-, three-, or four-years late initiators if they had a first statin dispensed between 366 and 1460 days after Tx.

In our analysis, age always refers to the age of the patient at the time of transplantation. We initially divided the study population into three age groups of roughly similar sizes: 16–48, 49–62, and over 62 years. International Guidelines recommend treating all adult KTRs (excluding fertile/pregnant females) with statins; however, for patients under 30, the treatment should be individualized [15]. We therefore decided to split the youngest age group into 16–29 and 30–48 years. When analyzing late initiators, we split the patients into two age groups <40 and ≥40 years, respectively.

### 2.4. Statin Use One Year before and One Year after Tx

The proportions of previous-, recurrent-, new-, and non-users in the study population were computed by sex and age group (16–29, 30–48, 49–62 and over 62 years). Statin use the year before and the year after Tx, was compared for patients receiving either CsA or Tac the first year after Tx. Use was defined as having filled at least one prescription of the respective drug. To identify if there have been variations in statin prescribing practices over time, statin use by KTRs during the year after Tx was studied in accordance with the year of transplantation. The proportion of Tx patients receiving statins in the first year after transplantation was compared in each of the 19 Norwegian counties (with populations ranging from 76,149 to 666,759) (January 2017). 

### 2.5. Adherence to Treatment and Treatment Intensity

Adherence to statin treatment was analyzed in a three-year follow-up for new and recurrent users after Tx. Only those who received the first kidney transplant in the period 2005–2013 were included in this analysis. The proportion of two- and three-years adherent users among those still alive at the end of the 2nd and 3rd year was computed separately for new and recurrent users. Treatment intensities of statins for the three years adherent users were measured as the mean annual amount dispensed in the number of DDDs per user in the 2nd and 3rd year after Tx. The DDD of fluvastatin was set to 60 mg based on the recommended dose used in the general adult population [18]. The annual number of treatment days covered was estimated from the mean annual amount of DDDs dispensed, assuming a daily dose of 80 mg of fluvastatin.

### 2.6. Initiation of Statins 2, 3 and 4 Years after Tx

We followed the patients with no statin dispensed the first year after Tx to see if they initiated statin treatment later (i.e., filled at least one prescription during the second, third or fourth year after transplantation). Only patients receiving a kidney transplant for the first time in the period 2005–2012 were included in this analysis. The cumulative proportions of late initiators of statins two, three, and four years after Tx (i.e., four years after includes all KTRs with at least one prescription dispensed between the in the 2–4-year period after Tx) were calculated among those still alive 2, 3 and 4 years after Tx for the age groups <40 years and ≥40 years.

### 2.7. Ethical Considerations

All individuals in the Norwegian Renal Registry have given their written informed consent to link the registry data with national health registries (including NorPD). The local ethical committee evaluated the project as a quality analysis and approval from the Norwegian Data Inspectorate was given as it was required to link the two databases.

## 3. Results 

### 3.1. Statin Use One Year before and after Tx

A total of 2492 patients (2418 > 16 years) received their first kidney Tx at Oslo University Hospital, Rikshospitalet in the period 2005–2015. Of the 2418, 81 died within one year after Tx and 61 experienced graft loss during the first year. Of the remaining patients, 26 were not registered in the NorPD during the first year after Tx as they were included in clinical trials supplying all immunosuppressant medications. Thus, a total of 2250 patients were included in the analysis, 69% men and 31% women. 

Patient age at Tx ranged from 16 to 83 years, with a mean age of 54 years and an interquartile range of 43–65 years. There was a high rate of pre-emptive first kidney Tx in the study period (27%) and the median time in dialysis before the first kidney Tx was 17 months. The numbers remained stable during the study period.

The proportion receiving Tac the year after Tx was 21% in 2005, 30% in 2006, between 48% and 55% from 2007–2011, 95% in 2012, and 99–100% between 2013–2015.

**Figure 1:**
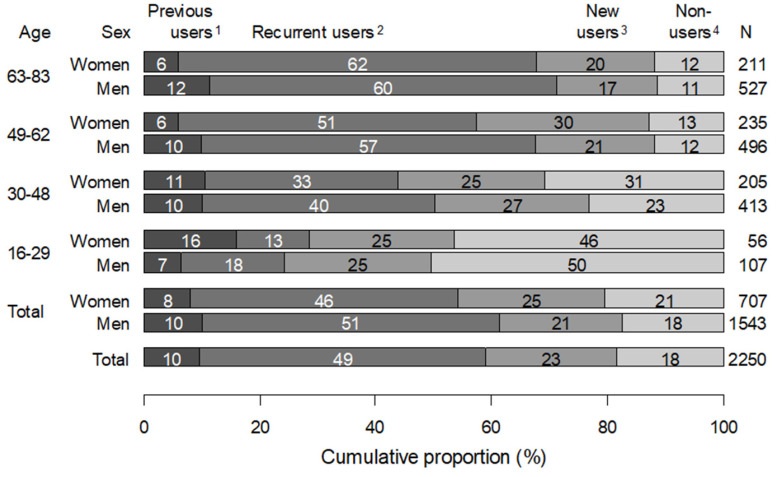
Proportion (%) of patients with statins dispensed one year (1–365 days) after and/or one year (364–0 days) before the first kidney transplant (Tx) in total and according to sex and age group. The number of KTRs is given in the right margin. ^1^ Use before but no use after Tx; ^2^ Use both before and after Tx; ^3^ No use before but use after Tx; ^4^ No use before or after Tx.

A total of 1620 KTRs (72%) received statins the first year after Tx. Of these, 31% (*n* = 508) were new users and 69% (*n* = 1112) were recurrent users. Six hundred thirty (28%) received no statins the first year after Tx. Of these, 66% (*n* = 413) were non-users and 34% (*n* = 217) were previous users. The number of statin users increased with age but were similar in men and women (Figure 1). In the age group 16–29 years, 59% (163 patients) received no statins the first year after Tx (62% of women and 57% of men), while in the age groups 49–62 and ≥ 62 years, 18 and 23% received no statins after Tx, respectively.

Of the new users of statins (23%) (*n* = 508), 97% were dispensed fluvastatin. Among the 1112 patients receiving statins both the year before and after Tx (recurrent users), 84% switched from another statin prescribed before Tx (mainly simvastatin or atorvastatin) to fluvastatin after Tx, while 12% used fluvastatin both before and after Tx. Of the remaining 4% of patients not using fluvastatin after Tx, 73% (*n* = 30) continued with the same statin they had used before, mainly simvastatin or atorvastatin. Among patients using statins other than fluvastatin the year after Tx (54 patients), the majority were on Tac (56%) or CsA only, whereas a small number (<10%) switched between the two calcineurin inhibitors.

**Figure 2:**
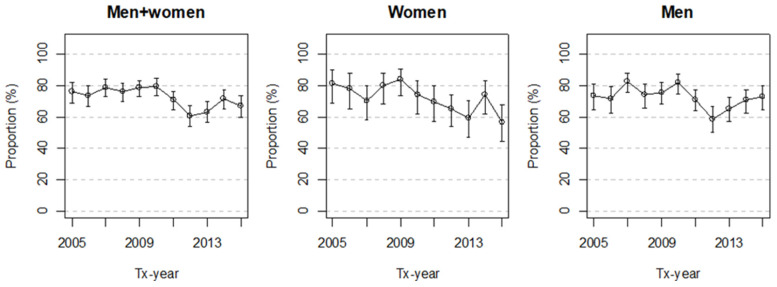
Proportion (%) of kidney transplant recipients according to year of transplantation (2005–2015) with statins dispensed in the first year after transplantation in total, and according to sex (with 95% confidence intervals). Tx—transplantation.

The proportion of the KTRs receiving statins in the first year after Tx varied according to year of transplantation, with a drop around 2011 (Figure 2). In the period 2005–2010 the proportion of statin users was on average 77%, whereas in 2012–2015 it was 66%. In women, the corresponding proportions were 78% and 63%, while in men they were 76% and 67% (Figure 2).

We found differences between the 19 counties in the proportions of Tx patients >16 years old receiving statins the first year after transplantation. The proportion varied from 60% in the county with lowest statin use (Aust-Agder) to 86% in the county with highest statin use (Sogn og Fjordane). There were no differences in the mean age of KTRs between these two counties (57 years) and the proportion of men was 74% in Aust-Agder and 61% in Sogn og Fjordane. 

### 3.2. Adherence to Treatment and Treatment Intensity

**Figure 3:**
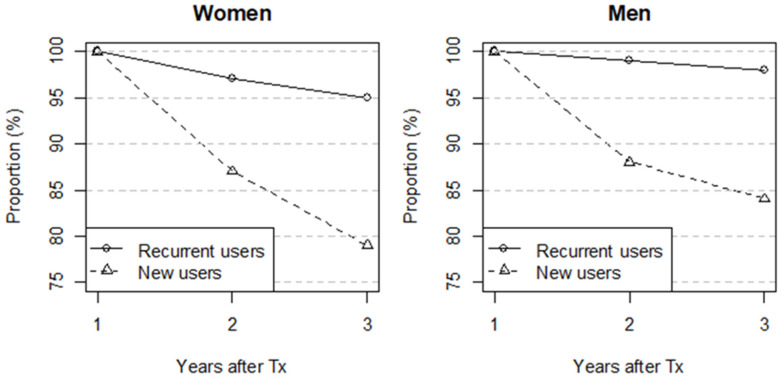
Adherence to statin treatment measured as the proportion (%) of new and recurrent users who had at least one statin dispensed the first year (year 1) after transplantation, according to sex. Year 2 includes those using statins both the first and second year, and year 3 includes patients using statins all three years after transplantation. The denominator is those still alive 2 and 3 years after transplantation, respectively. Tx–transplantation.

Among new users of statins in the period 2005–2013 (423 patients), 82% of the patients were adherent users both the second and third year after Tx, (84% of men and 79% of women), while the corresponding figure for recurrent users (926 patients) was 97% (98% of men and 95% of women) (Figure 3).

For patients using statins after Tx, both new and recurrent users were dispensed on average a similar annual amount of statins, measured in DDDs (473 and 474 DDDs in recurrent users in year 2 and 3 after Tx and 464 and 465 DDDs in new users, respectively). Based on the amount of statin dispensed, the estimated average number of treatment days during a year was 355 days for recurrent users and 349 days for new users.

### 3.3. Initiation of Statins 2, 3 and 4 Years after Tx

**Figure 4:**
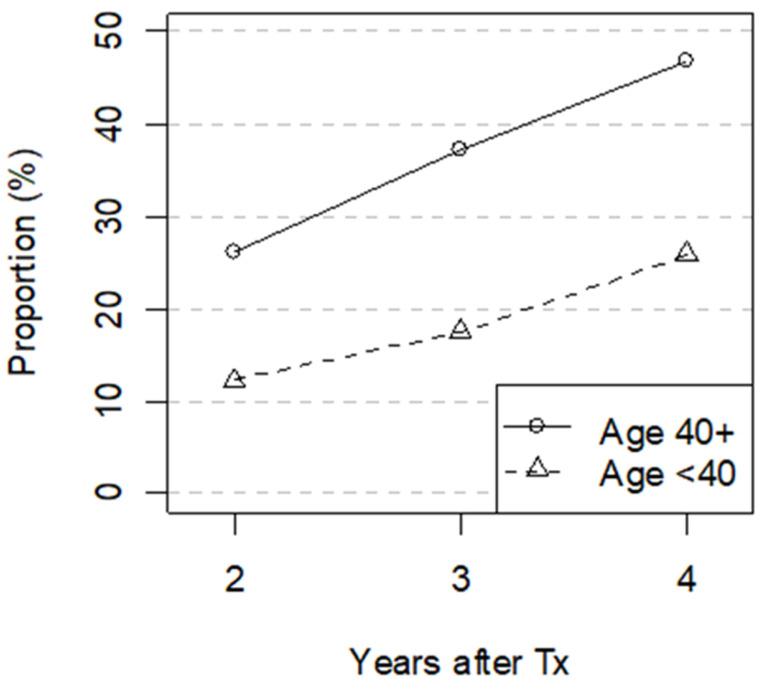
Proportion (%) of patients with no statin dispensed the first year after transplantation who initiated with statins within 2-, 3- and 4 years after Tx in the age groups <40 and ≥40 years. The denominator is those still alive 2, 3 and 4 years after transplantation, respectively. Tx–transplantation.

Of the 1639 patients who had the first Tx in 2005–2012, 429 patients did not receive a statin the first year after Tx (280 of 1325 aged ≥40 and 149 of 314 aged <40). Among patients over 40 years that were still alive two years after Tx, 26% had received a statin by the end of the second year and 47% had been prescribed a statin by the end of the fourth year. In the group under 40 years of age, 12% and 26% of those still alive received a statin by the end of the second and the fourth year, respectively (Figure 4). 

Of the 1467 patients who had the first Tx between 2005–2012 and were alive four years after Tx, 250 (17%) did not receive a statin the first four years after Tx.

## 4. Discussion

We found that 72% of kidney transplant recipients had a statin dispensed the first year after transplantation in the period 2005–2015 and the proportion was similar in men and women. Compared to other reports, our target achievement shows that we generally treat a higher proportion of our patients with statins [19,20]. 

The proportion of users was lowest in the youngest age group (16–29 years), which is probably due to individual CVD risk assessments performed on younger patients before prescribing a statin and/or due to statin contraindications in female recipients in this age category planning a pregnancy [21]. A slightly decreasing proportion of KTRs received statins in the most recently investigated years and this trend is more pronounced among women than men. During the study period, the prevalence of statin users in the general Norwegian population increased from 7–10% [17].

The proportion of statin users the first year after Tx differed across the Norwegian counties from 60% to 86%. Inter-county differences in prevalence of medicine use are common in all pharmacological classes and thus the variation in our study was not unexpected. Since our study includes a well-defined population, one could argue that the proportion of statin use should be more evenly distributed. The variation could be due to differences in perceptions about statin treatment held among prescribing specialists. A previous study comparing age distribution as it relates to inter-county differences of statin use in the general population in Norway, concluded that age does not account for the variations in statin use [22]. 

Our analysis shows that 26% of patients over 40 years not prescribed statin the first year after Tx received a statin during the second year. This number increased to 47% during a 2–4-year period. The corresponding proportions in KTRs under 40 years are 12% and 26%. The guidelines recommend statin use irrespective of plasma lipid levels so this late initiation of statins should not be due to increase in serum lipid levels detected late after Tx. It is possible that the treating physician has been afraid of calcineurin inhibitor-statin interactions and therefore postponed the start [23]. 

The proportion of statin users after Tx who discontinued statin treatment during the three years period after Tx was higher among new users (18%) than in recurrent users (3%). The higher adherence of statin treatment among recurrent users was expected, since these patients are known to tolerate statins. For the new users of statins it is expected that a part of them will experience side effects or intolerance and discontinue treatment. The adherence of statin use was slightly lower in new female users compared to men. Women seem to report more side effects from statins; this is being considered as a potential cause of differences across genders in the efficacy of statin use [24,25]. In addition, statins should not be used during pregnancy. All these factors may contribute to the observed gender differences in our study. The rate of adherence to statin treatments found in our study is higher than reported in other studies on statin use in the primary prevention of CVD [26,27]. 

The average amount of statins dispensed per user during one year showed that treatment intensity was equivalent to a daily use of 80 mg fluvastatin, the recommended dose in KTR patients. Similar figures were observed both in new and recurrent users of statins up to three years after Tx. Kidney transplant recipients are on a strict regime of immunosuppressant medications requiring life-long therapy to avoid graft loss and this will probably also influence statin use in a positive way, as observed in our study.

Of the patients receiving statins after Tx, use was in accordance with set guidelines and most patients were dispensed 80 mg of fluvastatin daily. 

Even though the overall percentage of patients using statins in our study was close to the 80% target set by the Norwegian Renal Registry, it was not anticipated that the rate of statin use would decrease during the investigated period. During this period there was also an increase in the number of patients using Tac. This cannot explain the decline in statin use, since there should be less clinically relevant interactions between Tac and the different statins. Dose reduction or switching immunosuppressive drugs can improve hyperlipidemia but very few practitioners implement this strategy to address hyperlipidemia. Use of mTOR inhibitors is known to increase lipid levels and the long-term CV effects remain unknown. KTRs require lifelong therapy with immunosuppressants and often other drugs, while facing an increased risk of complications arising from drug interactions. It has been debated whether statins should be recommended to all kidney transplant recipients. The lack of statistical significance in the primary endpoint of the ALERT trial and the fact that only one randomized trial has been made available could indicate the weak recommendation [15]. This is supported by a recent meta-analysis of statin use in chronic kidney disease patients, where it was concluded that statins are probably indicated in patients with a functioning transplant but that the number of KTRs investigated were too low for confident conclusions to be drawn [28]. Due to the price of conducting RCTs, additional clinical trials of lipid lowering treatments in KTRs are unlikely. Additionally, a recent large publication by Wyld et al. from the Australian and New Zealand Dialysis and Transplant Registry showed that occurrence rates of CV disease in KTRs still remained elevated but had been reduced by >40% in recent decades [29]. Unfortunately, their study had no information on patient statin use or plasma cholesterol levels. Real world evidence and patient-centered outcomes are increasingly important. Merging data from kidney transplant registries with long-term follow-up will probably be the best way to obtain proper data on the effect of statin therapies on CV outcomes. 

The strength of our study is that both the Norwegian Renal Registry and NorPD are nationwide registries, with close to 100% coverage, including data on transplantation and drug dispensing in primary care, respectively. We do not know whether medicines dispensed during this study were used, but the average amount dispensed during the year we examined indicates adherence to regular treatment over time. 

Our study population is too small and follow-up periods are currently too short to draw conclusions about the benefits of statin treatment in KTRs on endpoints such as total death, cardiac death and cardiovascular events compared to receiving no treatment with statins.

## 5. Conclusions

In conclusion, our findings show a slightly lower overall proportion of KTRs being prescribed a statin after transplantation than the national target level of 80%. The proportion of statin users increased with age but showed a decreasing trend in later time frames, both among women and men. Statin continuation rates were high.

## Data Availability

The data used in this study cannot be publicly shared, because of restrictions imposed by NorPD (Norwegian Institute of Public Health, Oslo, Norway). Researchers who wish to access the data should contact NorPD.
